# *Coptischinensis* Franch root rot infection disrupts microecological balance of rhizosphere soil and endophytic microbiomes

**DOI:** 10.3389/fmicb.2023.1180368

**Published:** 2023-05-25

**Authors:** Tao Tang, Fanfan Wang, Guobin Fang, Ting Mao, Jie Guo, Hui Kuang, Guangzhong Sun, Xiaoliang Guo, Yuanyuan Duan, Jingmao You

**Affiliations:** ^1^Key Laboratory of Chinese Herbal Medicine Biology and Cultivation, Ministry of Agriculture and Rural Affairs, Institute of Chinese Herbal Medicine, Hubei Academy of Agricultural Science, Enshi, China; ^2^Hubei Engineering Research Center of Good Agricultural Practices (GAP) Production for Chinese Herbal Medicines, Institute of Chinese Herbal Medicines, Hubei Academy of Agricultural Sciences, Enshi, China; ^3^Hubei Provincial Plant Protection Station, Wuhan, China

**Keywords:** *Coptis chinensis*, rhizosphere soil and endophytic microbiomes, microecological regulation, microecological balance, soil physicochemical factors

## Abstract

**Introduction:**

The ecological balance of the plant microbiome, as a barrier against pathogens, is very important for host health. *Coptis chinensis* is one of the important medicinal plants in China. In recent years, Illumina Miseq high-throughput sequencing technology was frequently used to analyze root rot pathogens and the effects of root rot on rhizosphere microorganisms of *C. chinensis*. But the effects of root rot infection on rhizosphere microecological balance of *C. chinensis* have received little attention.

**Methods:**

In this study, Illumina Miseq high-throughput sequencing technology was applied to analyze the impact on microbial composition and diversity of *C. chinensis* by root rot.

**Results:**

The results showed that root rot infection had significant impact on bacterial α-diversity in rhizome samples, but had no significant effect on that in leaf samples and rhizosphere soil samples, while root rot infection exhibited significant impact on the fungal α-diversity in leaf samples and rhizosphere soil samples, and no significant impact on that in rhizome samples. PCoA analysis showed that the root rot infection had a greater impact on the fungal community structure in the rhizosphere soil, rhizome, and leaf samples of *C. chinensis* than on the bacterial community structure. Root rot infection destroyed the microecological balance of the original microbiomes in the rhizosphere soil, rhizome, and leaf samples of *C. chinensis*, which may also be one of the reasons for the serious root rot of *C. chinensis.*

**Discussion:**

In conclusion, our findings suggested that root rot infection with *C. chinensis* disrupts microecological balance of rhizosphere soil and endophytic microbiomes. The results of this study can provide theoretical basis for the prevention and control of *C. chinensis* root rot by microecological regulation.

## 1. Introduction

*Coptis chinensis* is a perennial herb belonging to family Ranunculaceae, and genus *Coptis*. Its rhizome is one of the most commonly used materials in traditional Chinese medicine (He et al., 2017). The using of *C. chinensis* in traditional Chinese medicine has a long history, which can be traced back to the earliest known work on Chinese medicine in my country, Divine Farmer’s Materia Medica (Shennong Bencao Jing) (Liu et al., 2021). *C. chinensis* rhizome has broad antibacterial, antiviral, and antioxidant effects, and it is often used to treat dysentery, jaundice, acute fever-induced sore throat, fever, and diarrhea, and other diseases (Tseng et al., 2020). The latest research showed that the active substances in Huanglian Jiedu Decoction (toxicity-relief soup) can treat new coronavirus pneumonia through multiple targets and multiple pathways (Huang et al., 2020). Part of the mountainous areas above 1,200 meters above sea level in Enshi Tujia and Miao Autonomous Prefecture are one of the main *C. chinensis*-producing areas with a total planting area of about 5,000 hm^2^ and a total annual output of about 4,000 t. However, with the continuous expansion of the planting scale and continuous cultivation for many years, the root rot of *C. chinensis* occurred in Lichuan city and other places, resulting in a large-scale production reduction or even no harvest, which seriously decreasing the yield and quality of *C. chinensis* and adversely affecting the planting enthusiasm of farmers. *Fusarium avenaceum* and *Fusarium solani* have been reported to be pathogen of *C. chinensis* root rot disease (Luo et al., 2014; Mei et al., 2021b). *Diaporthe eres* has also been reported to cause *C. chinensis* root rot (Mei et al., 2021a). Our previous study has found that *Phoma aquilegiicola* and *Colletotrichum boninense* can cause rhizome rot of *C. chinensis*. There are many types of pathogens causing *C. chinensis* root rot disease making prevention and control of *C. chinensis* root rot difficult.

In animal disease theory, the “disease triangle” is often used to describe the dynamic interactions between hosts, pathogens, and environmental factors including microorganisms (Scholthof, 2007). This theory also applies to plants. The plant microbiome plays an indispensable role in various biological processes of the host. As the same, the microbial community in environment can affect various biological processes of the host and the host microbiome. Recent studies have shown that biological and environmental factors (including microorganisms) have a strong influence on host disease dynamics (Hawkes et al., 2020). Gut microbes, just like the skin, are the first barrier of immunity and play an important role in host defense against pathogens (Bernardo-Cravo et al., 2020). With the maturity of Illumina Miseq high-throughput sequencing technology, researchers began to pay more attention to the effect of pathogen infection on plant microbiome. Relevant studies have shown that the microbial community structure in plant rhizosphere soil, stem, and phyllosphere will change after infection by pathogen. The analysis of the soil fungal community structure in verticillium wilt disease-infected cotton fields used the Illumina Miseq high-throughput sequencing technology has shown that the number and abundance of fungal OTUs in severely diseased cotton fields were higher than those in mildly-diseased or disease-free fields, while the fungal diversity was reduced in diseased cotton fields (Liu et al., 2019). Zhang et al. have reported that the number of *Fusarium* and *Verticillium* in the rhizosphere soil of diseased cotton was higher, while the number of beneficial microorganisms such as *Trichoderma* in the rhizosphere soil of healthy cotton was significantly higher than that of diseased cotton (Zhang et al., 2022). Liao et al. (2021) quickly identify potential pathogenic and antagonistic microorganisms related to root rot of *C. chinensis* through high-throughput sequencing technology. The results indicated that *Fusarium*, *Volutella*, *Cladorrhinum*, *Cylindrocarpon*, *Cylindrocarpon* and *Exophiala* were potential pathogenic agents of *C. chinensis* root rot, and *Bacillus* may be potential antagonists. However, most of the previous studies focused on the influence of root rot infection on rhizosphere microbiome structure of *C. chinensis*, but little attention was paid to the influence on ecological balance. The destruction of the ecological balance of host microorganisms by pathogens may be an important reason for the difficulty of disease control. Microecological regulation refers to the purpose of preventing and controlling plant diseases by regulating the microbial environment of plants so that the microbial environment was beneficial to the host and not conducive to pathogens (Berendsen et al., 2012). Bai et al. (2015) isolated more than 400 culturable endophytic bacteria from the roots and leaves of *Arabidopsis thaliana*, and reconstructed the microbiome in the roots and leaves of *A. sterile* shoots, creating a research model for reconstructing the microbiome. Li et al. (2021) analyzed the characteristics of the microbial community in the rhizosphere and root of *Astragalus mongholicus* with root rot, and constructed a synthetic community based on significantly enriched bacteria in the rhizosphere of *A. mongholicus* with root rot. The synthetic community can inhibit the growth of pathogen and activate plant-induced systemic resistance of *A. mongholicus* to achieve the purpose of reducing the incidence of root rot (Li et al., 2021). Using the theory of microecology and the method of microecological regulation to adjust the microbial community of plants to improve their disease resistance can be an important research idea for the prevention of *C. chinensis* root rot.

In this study, Illumina Miseq high-throughput sequencing technology was used to analyze the composition and diversity of rhizomes, leaf endophytic microorganisms, and rhizosphere soil microorganisms of healthy and diseased *C. chinensis* from two different locations. The objective of this study was as followed: (1) to clarify the differences of rhizosphere soil, rhizosphere and leaf microbial community structure in *C. chinensis*; (2) to clarify the effects of *F. avenaceum* and *F. solani* infection on rhizosphere soil, rhizosphere and leaf microbial community structure; (3) to provide theoretical support for using micro-ecological control methods or SynComs to prevent and control *C. chinensis* root rot.

## 2. Materials and methods

### 2.1. Sampling site and sampling method

All the sequencing samples were collected from the *C. chinensis* Planting Base (108° 35′ E, 30° 22′ N) in Jianzhuxi Town, Lichuan City, Enshi Prefecture at an altitude of 1,300 m in October, 2019. The samples were collected from two different sites and divided into 12 groups as shown in Table 1. The samples were taken from 5 different positions in the same field ridge and mixed uniformly into one sample. The rhizosphere soil around 5–10 cm below the ground part of the *C. chinensis* plant was collected by using the JC-802 root soil sampler. The rhizome samples obtained from the same 5 positions of the rhizosphere soil samples and leaf samples were mixed uniformly into one sample. The rhizome samples and the leaf samples were surface-sterilized with 75% ethanol immediately after sampling. Three samples in each group were collected from different positions, and the experiments were conducted in three biological repetitions. The samples were put into sterile 50 ml centrifuge tubes, transported to the laboratory at low temperature, and stored at 4°C for later use. The distance between the two sampling sites was more than 3 km. *F. avenaceum* and *F. solani*, isolated and identified in both locations, were the main pathogens of root rot on *C. chinensis* from Lichuan City.

**Table 1:** The collection information of experimental samples.

Samples ID	Samples type	Collection site	Disease status
DJT1-3	Rhizosphere soil	Dadaojiao village	Health
DBT1-3	Rhizosphere soil	Dadaojiao village	Infection
DJG1-3	Rhizome	Dadaojiao village	Health
DBG1-3	Rhizome	Dadaojiao village	Infection
DJY1-3	Leaf	Dadaojiao village	Health
DBY1-3	Leaf	Dadaojiao village	Infection
BJT1-3	Rhizosphere soil	Banchangping village	Health
BBT1-3	Rhizosphere soil	Banchangping village	Infection
BJG1-3	Rhizome	Banchangping village	Health
BBG1-3	Rhizome	Banchangping village	Infection
BJY1-3	Leaf	Banchangping village	Health
BBY1-3	Leaf	Banchangping village	Infection

### 2.2. Analysis of physicochemical properties of rhizosphere soil

The soil physicochemical indexes were determined as follows. The deionized distilled water (1:20 w/v) was used to make soil suspension, and Mettler-Toledo FE 20 was used to measure soil pH. Soil organic matter (TOM) was determined using the potassium dichromate external heating method [38]. Soil total nitrogen (TCN) was detected by Kjeldahl determination (Bremner, 1960). Soil total phosphorus (TCP) was determined by the molybdenum antimony blue colorimetry (Murphy and Riley, 1962). Soil total potassium (TCK) was determined by flame atom absorption method (Wu et al., 2017). Soil available nitrogen (AN) was determined by diffusion absorption method (Michrina et al., 1982). Soil available phosphorus (AP) was analyzed following the methods described by Li et al., (2020). Soil available potassium (AK) was determined by atomic absorption spectrometry (Tian et al., 2021). Soil physicochemical factors were determined by the Institute of Plant Protection and Soil and Fertilizer of Hubei Academy of Agricultural Sciences.

### 2.3. DNA extraction and PCR amplification

The total microbial DNA of rhizosphere soil samples was extracted with PowerSoil® DNA Isolation Kit (MOBIO, United States). The rhizomes and leaves were chopped and ground with liquid nitrogen, and the total DNA was extracted by PowerSoil® DNA Isolation Kit. The DNA concentration and purity were determined by Nanodrop ultraviolet spectrophotometer (Thermo Scientific, United States). The quality of DNA extract was detected by 1.2% agarose gel electrophoresis, and then the DNA extraction solution was stored at −80°C for later use. The V5-V7 region of fungal 18S rRNA was amplified by using universal primers SSU0817F (5’-TTAGCATGGAATAATRRAATAGGA-3′) and 1196R (5’-TCTGGACCTGGTGAGTTTCC-3′; Jiang et al., 2017). The V3-V4 region of 16S rRNA was PCR amplified by using universal primers 338\u00B0F (5’-ACTCCTACGGGAGGCAGCA-3′) and 806 R (5’-GGACTACHVGGGTWTCTAAT-3′; Li et al., 2020). Pfu high-fidelity DNA polymerase from TransGen Biotech was used for PCR amplification with the number of amplification cycles strictly controlled to make cycle number as small as possible and the amplification conditions for the same batch of samples consistent. The PCR products were purified and recovered with the AxyPrep DNA Gel extraction kit. Subsequently, each sample was quantified using BioTek Flx800 microplate reader with Quant-iT PicoGreen dsDNA Assay Kit (Invitrogen, P7589).

### 2.4. Library preparation and on-board sequencing

Sequencing libraries were prepared using the TruSeq Nano DNA LT Library Prep Kit from Illumina. After passing the test, the constructed library was sequenced by Shanghai Parsenor Biotechnology Co., Ltd. using the Illumina Miseq platform. Before computer sequencing, it is necessary to adopt Agilent High Sensitivity DNA Kit to conduct quality of constructed library inspection on Agilent Bioanalyzer. After that, Quant-iT PicoGreen dsDNA Assay Kit was used to quantify the library on the Promega QuantiFluor system, and the qualified concentration of the library should be above 2 nM. The qualified computer sequencing libraries were diluted in gradient, mixed in proportion to the required sequencing volume, and denatured into single strand by NaOH for computer sequencing. All raw data has been uploaded to GSA with the GSA number: PRJCA008804.^1^

### 2.5. Sequencing data processing

For the raw data obtained from sequencing, firstly, qiime cutadapt trim-paired command was executed to excise the primer fragments of the sequence. The unmatched primer sequences were discarded, and then the qiime DADA2 denoise-paired command was executed for quality control, denoising, splicing, and chimera clearance (Bolyen et al., 2019). The above steps were performed separately for each library. After all the libraries were denoised, the ASVs feature sequences and ASV tables were merged, and singletons ASVs were removed. The classify-sklearn algorithm in QIIME2^2^ with default parameters was adopted to preform species annotation for the feature sequence of each ASV using a pre-trained Naive Bayes classifier (Bokulich et al., 2018). The qiime feature-table rarefy function in the QIIME2 software was employed to subsample all samples with subsampling depth set as 95% of the minimum sample sequence size. For bacteria 16 s rRNA genes, the Greengenes database (Release13.8^3^) was selected by default. For fungal ITS sequence, the UNITE database (Release 8.0^4^) was selected by default.

### 2.6. Statistical analysis

Statistical analysis was performed using SPSS software (versions 18.0) and R (version 4.0.2). Principal Component Analysis (PCoA) was performed on “the Bray–Curtis distances” using the R package VEGAN (version 2.5–7) package (Bray and Curtis, 1957). The PCoA plots were compared using the procrustes analyzes in the VEGAN. Venn plots were generated using the R package ‘VennDiagram’ (version 1.6.20) (Zaura et al., 2009). Differential core microorganisms in each sample were statistically analyzed using the R software package “DESeq2 version 1.28.1.” The correlation network between core microorganisms and the correlations between core microorganisms and soil physicochemical factors were analyzed by using Spearman algorithm in R package WGCNA (version 1.69) and the network diagram was drawn by Gephi 0.9.2 (Langfelder and Horvath, 2008). GraphPad Prism software (version 7.0) was used for statistical analysis and data plotting. Significant differences among microbial diversity were analyzed using *t*-test in GraphPad Prism software (version 7.0). The level of significance was set at *p < 0.05 and **p < 0.01.

## 3. Results

### 3.1. Soil physicochemical properties

As shown in Table 2, the pH of healthy *C. chinensis* rhizosphere soil in two different locations was significantly higher than that of root rot *C. chinensis* rhizosphere soil (*p* < 0.05). In addition, the rhizosphere soil organic matter, alkali-hydrolyzed nitrogen, available phosphorus, total phosphorus, and total nitrogen of healthy *C. chinensis* rhizosphere soil were significantly higher than those of root rot *C. chinensis* rhizosphere soil (*p* < 0.05). However, the contents of available potassium and total potassium were inconsistent in the two different locations. In the samples from Daojiao Village, the content of available potassium in root rot *C. chinensis* rhizosphere soil was significantly higher than that in the healthy *C. chinensis* rhizosphere soil (*p* < 0.05), whereas the content of total potassium was not significantly different. The contents of available potassium and total potassium in the root rot *C. chinensis* rhizosphere soil samples from Banchangping Village were significantly lower than those in the healthy *C. chinensis* rhizosphere soil samples (*p* < 0.05). This indicated that soil pH, organic matter, alkali-hydrolyzed nitrogen, available phosphorus, total phosphorus, and total nitrogen content may have a certain relationship with the occurrence of *C. chinensis* root rot.

**Table 2:** Physiochemical characteristics of rhizosphere soil of healthy and infected *Coptis chinensis*.

Samples ID	pH	TOM	AN	AP	AK	TCN	TCP	TCK
		g/kg	mg/kg	mg/kg	mg/kg	%	%	%
DBT	4.73 ± 0.025d	17.94 ± 0.688b	64.74 ± 1.335d	105.23 ± 2.147b	146.95 ± 0.738c	0.12 ± 0.005b	0.11 ± 0.001b	2.29 ± 0.043a
DJT	4.80 ± 0.021c	19.11 ± 0.203a	71.85 ± 3.359c	137.96 ± 0.941a	128.23 ± 1.224d	0.13 ± 0.002a	0.15 ± 0.001a	2.23 ± 0.036a
BBT	4.91 ± 0.021b	15.81 ± 0.341c	87.43 ± 1.541b	31.04 ± 0.332d	151.21 ± 0.738b	0.12 ± 0.002b	0.05 ± 0.001d	2.08 ± 0.010c
BJT	5.18 ± 0.025a	18.27 ± 0.611ab	152.39 ± 3.359a	70.13 ± 1.657c	217.72 ± 1.279a	0.14 ± 0.002a	0.06 ± 0.001c	2.12 ± 0.020b

TCN, total content of nitrogen; TCP, total content of phosphorus; TCK, total content potassium; TOM, total organic matter; NA, available nitrogen; NP, available phosphorus; NK, available potassium. Values are means ± standard deviation (*n* = 3). Lower-case letters (a, b, c, d) for a given factor indicate significant difference (*p* < 0.05; One-way ANOVA test in GraphPad Prism). DJT, Rhizosphere soil samples of healthy Coptis chinensis in Dadaojiao Village; DBT, Rhizosphere soil samples of Coptis chinensis infected with root rot in Dadaojiao Village; BJT, Rhizosphere soil samples of healthy Coptis chinensis in Banchangping Village; BBT, Rhizosphere soil samples of Coptis chinensis infected with root rot in Banchangping Village. The same below.

### 3.2. Alpha diversity

**Figure 1:**
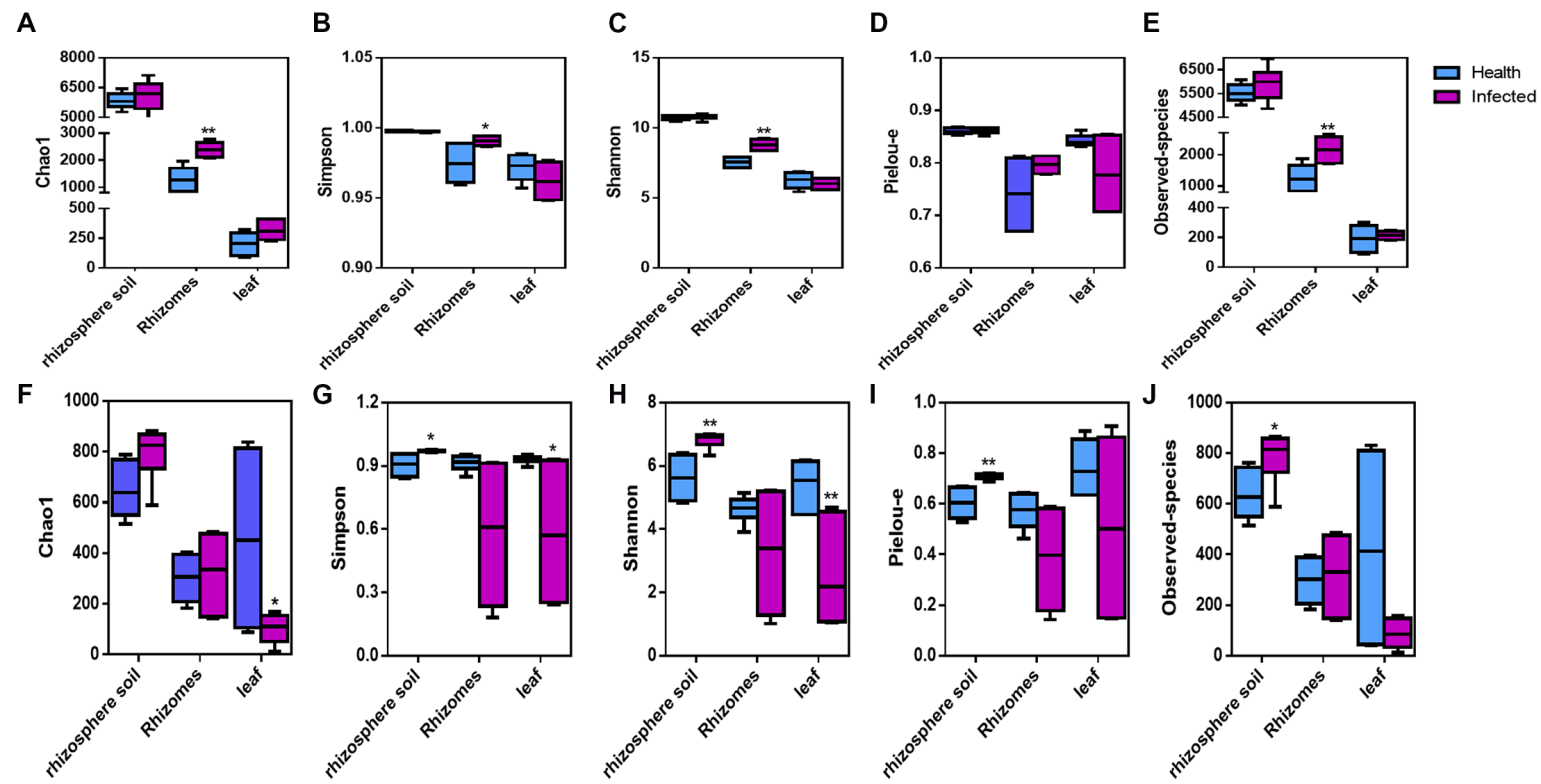
Alpha diversity indices of the bacteria and fungal community in rhizosphere soils, rhizome, and leaf of *C. chinensis.* **(A)** Chao1 index of the bacteria community in all samples; **(B)** Simpson diversity index of the bacteria community in all samples; **(C)** Shannon diversity index of the bacteria community in all samples; **(D)** Pielou evenness of the bacteria community in all samples; **(E)** Observed species of the bacteria community in all samples; **(F)** Chao1 index of the fungi community in all samples; **(G)** Simpson diversity index of the fungi community in all samples; **(H)** Shannon diversity index of the fungi community in all samples; **(I)** Pielou evenness of the fungi community in all samples; **(J)** Observed species of the fungi community in all samples. ‘*’ and ‘**’ represent significant differences between health and infected of rhizosphere soils, rhizome, and leaf of *C. chinensis*, respectively (Meltiple *t*-tests-one per row in GrahPad Prism).

The α-diversity indices Chao1, Shannon, Simpson, Observed-species, and Pielou-e were used to evaluate the diversity and richness of each sample. The Observed species index referred to the number of OTUs actually contained in the sample, while the chao1 index referred to the number of OTUs estimated in the sample, both of which reflected the number of OTUs (species) in the sample. Shanon index was used to describe the disorder and uncertainty in OTUs. Simpson index referred to the probability of taking two sequences randomly from the sample, which belong to different OTUs. Shannon and Simpson index not only reflect the number of species in the sample (richness) but also reflect the abundance distribution of each species in the sample (evenness). The group analysis of healthy *C. chinensis* and those infected with root rot disease was performed. As shown in Figure 1, the infection with root rot disease significantly increased the α-diversity index including Chao1, Shannon, Simpson and Observed-species in *C. chinensis* rhizome samples, but it had no significant effect on the α-diversity index in the rhizosphere soil samples and leaf samples of *C. chinensis*. As for fungal α-diversity, the infection with root rot disease significantly increased the α-diversity indices including Simpson, Shannon, Observed-species, and Pielou-e in the rhizosphere soil samples of *C. chinensis*, but significantly decreased Simpson and Shannon indices in *C. chinensis* leaf samples, and the infection had no significant effect on the α-diversity index in *C. chinensis* rhizome samples. The effects of root rot infection on the bacterial and fungal community structure in *C. chinensis* rhizosphere soil, rhizome, and leaf samples exhibited opposite trends. Namely, root rot infection had the greatest effect on the bacterial alpha diversity of rhizome samples, but no effect on leaf and rhizosphere soil samples. However, root rot infection had the greatest effect on the fungal alpha diversity of leaf samples and rhizosphere soil samples, but the least effect on the rhizome samples. In addition, in terms of the Chao1 and Observed-species indices of bacterial α-diversity, the bacterial abundance in the *C. chinensis* rhizosphere soil samples were significantly higher than that in the rhizome samples, which in turn was significantly higher than that in the leaf samples. However, the fungal abundances were not significantly different.

### 3.3. β diversity

**Figure 2:**
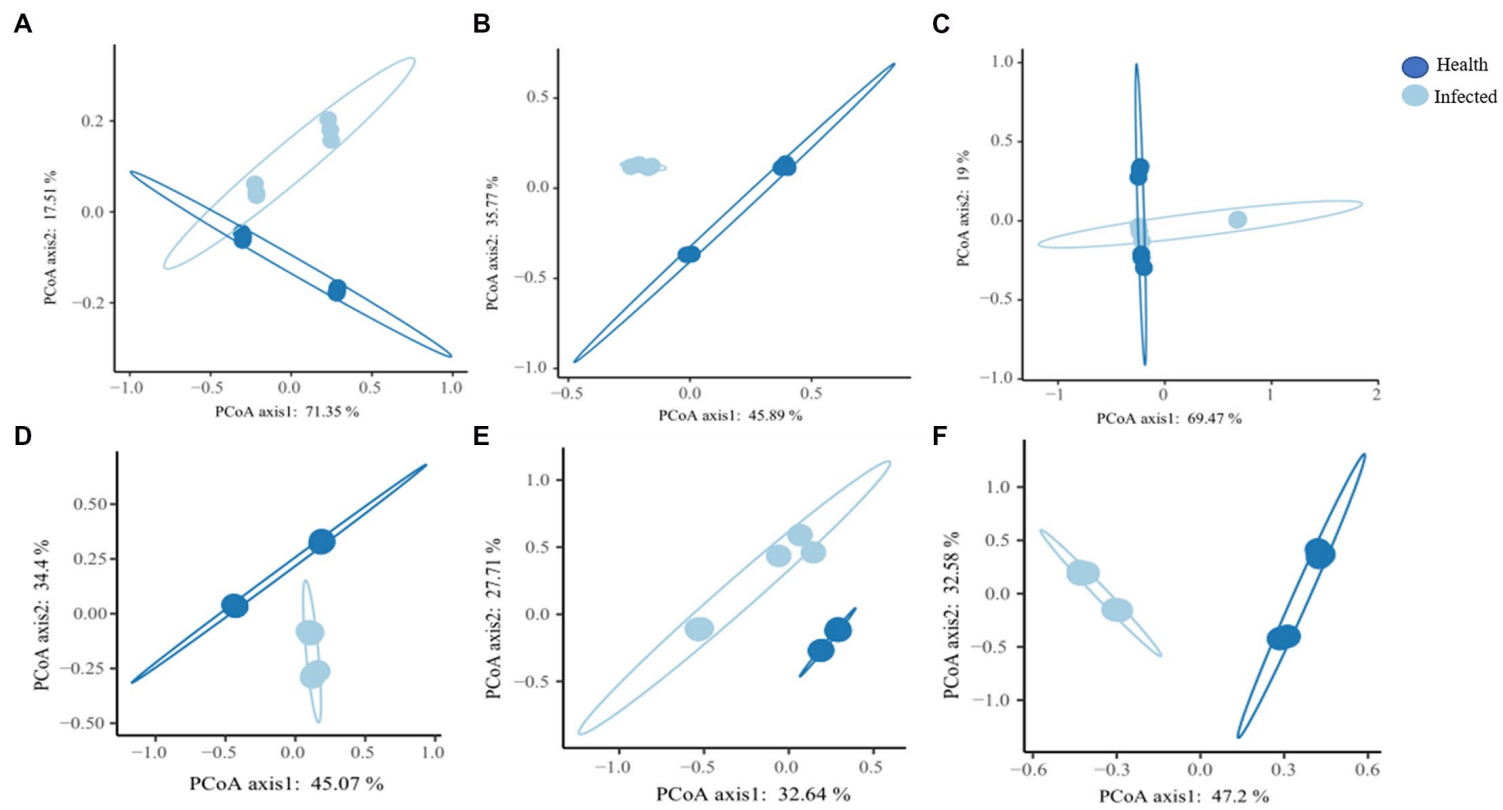
Principle coordinate analysis (PcoA) of bacteria and fungal community in rhizosphere soils, rhizome, and leaf of health and infected *C. chinensis*. **(A)** Principle coordinate analysis (PcoA) of bacteria community in rhizosphere soils of bacteria of health and infected *C. chinensis*; **(B)** Principle coordinate analysis (PcoA) of bacteria community in rhizome of health and infected *C. chinensis*; **(C)** Principle coordinate analysis (PcoA) of bacteria community in leaf of health and infected *C. chinensis*; **(D)** Principle coordinate analysis (PcoA) of fungal community in rhizosphere soils of bacteria of health and infected *C. chinensis*; **(E)** Principle coordinate analysis (PcoA) of fungal community in rhizome of health and infected *C. chinensis*; **(F)** Principle coordinate analysis (PcoA) of fungal community in leaf of health and infected *C. chinensis*.

β diversity differences between healthy group and root rot infection group samples were analyzed using Bray-Curtis distance matrix. As shown in Figure 2, bacterial communities in healthy rhizome samples and those infected with root rot were clustered separately, with the first two axes explaining 45.89 and 35.77% of the total variance (Figure 2B). The bacterial communities of healthy *C. chinensis* and root rot-infected *C. chinensis* rhizosphere soil and leaf samples exhibited a certain intersection in the PCoA map (Figures 2A,C). In contrast, the fungal communities in the rhizosphere soil, rhizome, and leaf samples of healthy *C. chinensis* and root rot disease-infected *C. chinensis* were all clustered separately (Figures 2D–F). This result showed that root rot disease had a great impact on the fungal community structure of the rhizosphere soil, rhizome, and leaf samples of *C. chinensis*, and it had a great impact on the bacterial community of the rhizome samples of *C. chinensis*, but less impact on the bacterial community of rhizosphere soil and leaf samples. This result was consistent with that of alpha diversity.

### 3.4. Analysis of bacterial and fungal community structure

At the phylum level, the main bacterial communities in the rhizosphere soil, rhizome, and leaf samples of *Coptis chinensis* were Proteobacteria, Acidobacteria, Actinobacteria, Chloroflexi, and Bacteroidetes. The main fungal communities in the rhizosphere soil, rhizome, and leaf samples of *C. chinensis* were Ascomycota and Basidiomycota.

**Figure 3:**
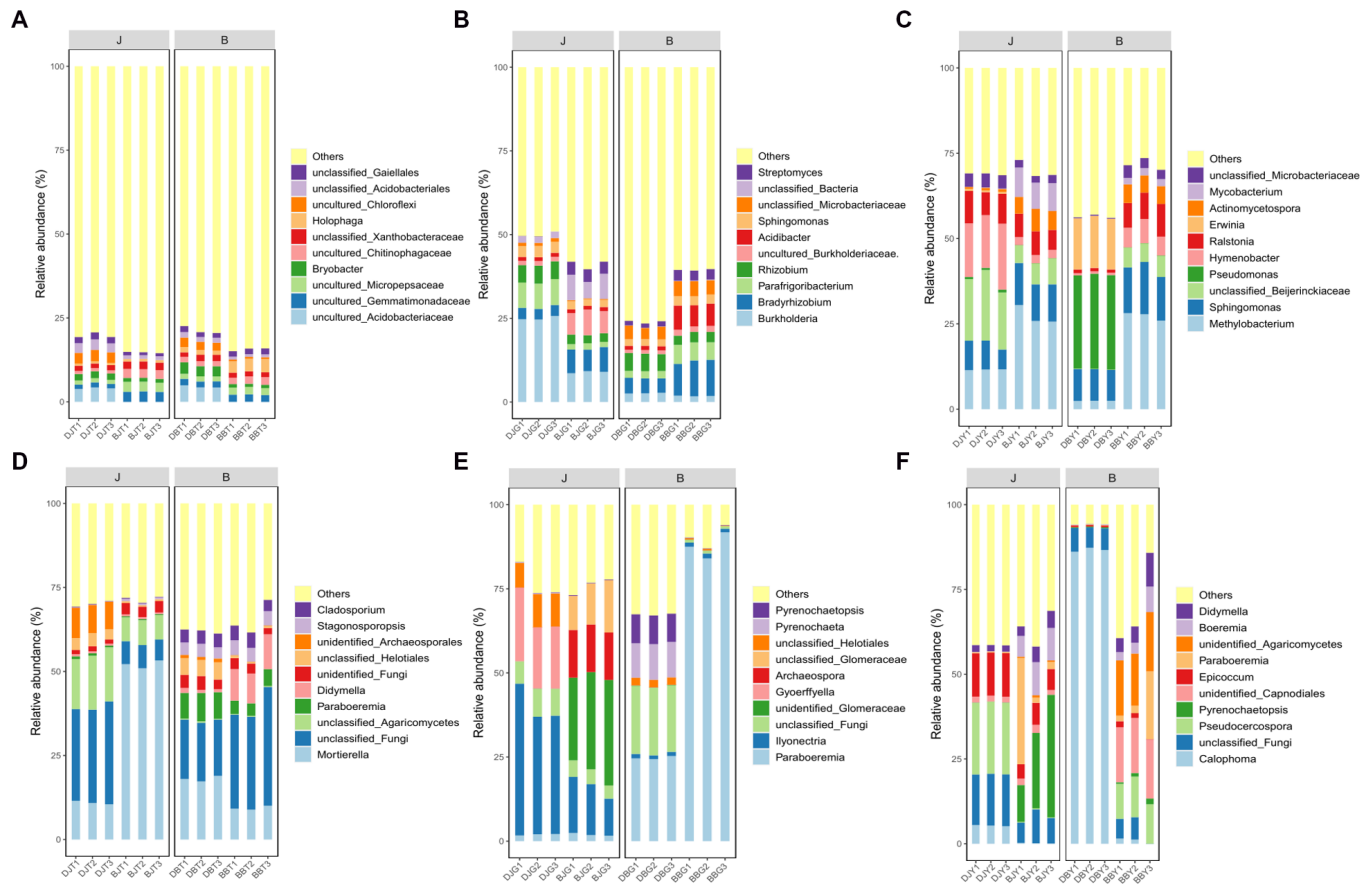
Bacterial and fungus composition in rhizosphere soils, rhizome, and leaf of health and infected *C. chinensis* at the phylum level. **(A)** Bacterial composition in rhizosphere soils of health and infected *C. chinensis* at the phylum level; **(B)** Bacterial composition in rhizome of health and infected *C. chinensis* at the phylum level; **(C)** Bacterial composition in leaf of health and infected *C. chinensis* at the phylum level; **(D)** Fungus composition in rhizosphere soils of health and infected *C. chinensis* at the phylum level; **(E)** Fungus composition in rhizome of health and infected *C. chinensis* at the phylum level; **(F)** Fungus composition in leaf of health and infected *C. chinensis* at the phylum level.

At the genus level, the fungal and bacterial community compositions from the different samples (rhizosphere soil, rhizome, and leaf) were quite different. The sequencing results showed that the dominant bacterial genera in the rhizosphere soil samples of *C. chinensis* were *Holophaga* and *Bryobacter,* but most others were not annotated at the genus level (Figure 3A). The dominant bacterial genera in the rhizome samples of *C. chinensis* were *Burkholderia, Bradyrhizobia, Parafrigoribacteria,* and *Rhizobia.* The infection with root rot disease significantly suppressed the relative abundance of *Burkholderia*. *Bradyrhizoloium*, *Acidibacter*, and *Streptemyces* were significantly enriched in the diseased rhizome samples (Figure 3B). The dominant bacterial genera in *C. chinensis* leaf samples were *Methylobacteria* and *Sphingomonas* (Figure 3C). *Mortierella* and a type of unannotated fungus were dominant fungal communities in the rhizophere soil samples of *C. chinensis* (Figure 3D). The dominant fungal genera of the healthy *C. chinensis* rhizome samples and the diseased *C. chinensis* rhizome samples were completely different. The dominant fungal community genera of the healthy *C. chinensis* rhizome samples were *Ilyonectria*, while that of the diseased *C. chinensis* rhizome samples was *Paraboeremia* (Figure 3E). The dominant bacteria in the fungal community of healthy *C. chinensis* leaf samples and diseased *C. chinensis* leaf samples were quite different. The dominant fungal genus in the fungal community in healthy *C. chinensis* samples from Daojiao Village was *Pseudocercospora* and that *from* Banchangping Village was Pyrenochaetopsis, while the dominant fungal genus in the infected *C. chinensis* leaf samples collected from Daojiao Village was *Calophoma*, and the dominant fungal community genera in the infected *C. chinensis* leaf samples collected from Banchangping Village were unidentified *Agaricomycetes* and unidentified*_Capnodiales* (Figure 3F). The influence of root rot infection was greater on the fungal community structure of various *C. chinensis* samples than on the bacterial community structure, implying that *C. chinensis* root rot was mainly caused by fungal pathogens.

### 3.5. Overall association analysis of fungal and bacterial microbiomes

**Figure 4:**
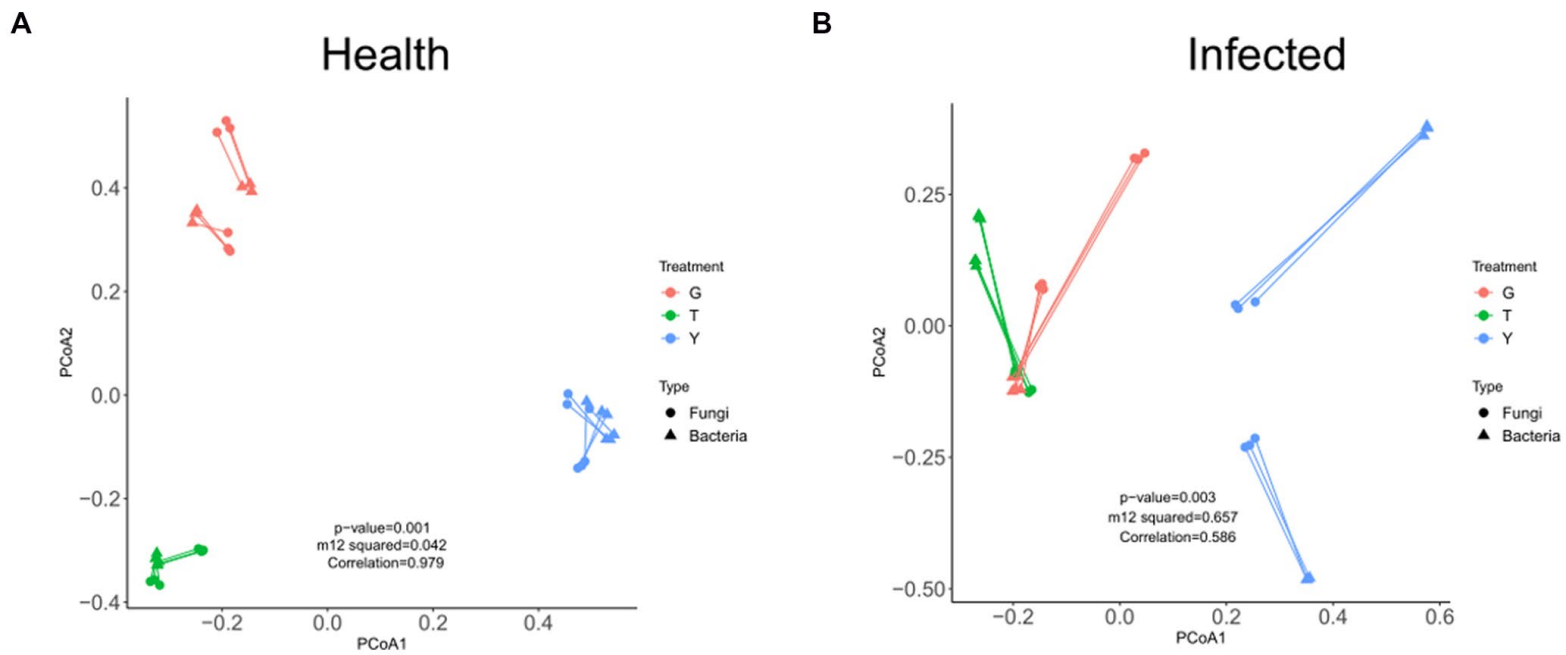
Procrustes analysis under fungal microbiome and bacterial microbiome of health and infected samples. **(A)** Procrustes analysis under fungal microbiome and bacterial microbiome of health samples; **(B)** Procrustes analysis under fungal microbiome and bacterial microbiome of infected samples. The color of each dot/triangle represents fungal microbiome/bacterial microbiome. Satisfying conditions were significant (m12^2^ values of <0.45, correlation values of >0.775, and significance values of ≤0.001).

The overall correlation between fungal and bacterial microorganisms in healthy *C. chinensis* samples and diseased *C. chinensis* samples was analyzed by procrustes analysis. As shown in Figure 4, the overall structure between bacterial microbiome and fungal microbiome in healthy *C. chinensis* rhizosphere soils, rhizome, and leaf samples was significantly correlated (m12^2^ values <0.45, correlation values >0.775, and significance values ≤0.001; Figure 4A). However, the overall structural correlation between bacterial microbiome and fungal microbiome in the rhizosphere soil, rhizome, and leaf samples of *C. chinensis* disappeared after root rot infection (Figure 4B). This result indicated that root rot infection of *C. chinensis* severely damaged the ecological balance of the original bacterial microbiome and fungal microbiome of healthy *C. chinensis*, and root rot infection significantly inhibited the interaction of endophytic fungi and bacteria in *C. chinensis*.

Core microbes refer to those whose relative abundance are greater than 0.05% and who are present in at least half of the samples. In this study, an interaction network analysis was performed of the core microbes of fungi and bacteria in healthy and diseased *C. chinensis* samples. Sixty core microbes were identified from healthy *C. chinensis* rhizosphere soil, rhizomes, and leaf samples, including 26 fungi and 34 bacteria (Supplementary Table S1). A total of 60 core microbes were identified from the infected *C. chinensis* samples, including 38 fungi and 22 bacteria (Supplementary Table S2). Based on the R package WGCNA, the Spearman algorithm was used to analyze the correlation network between the healthy and diseased core microbes. The results showed that the fungal microbes in the healthy *C. chinensis* samples were significantly separated from bacterial microbes, and the bacterial distribution was denser and the correlation was more complex (Supplementary Figure S1A). The independent effects between bacteria and fungi in the infected *C. chinensis* samples were disrupted, and the correlation of fungi was also significantly weakened, while bacterial correlation network became denser (Supplementary Figure S1A). This result corroborates with the results of Procrustes analysis.

### 3.6. Microbial difference analysis of healthy and diseased *Coptis chinensis* samples

**Figure 5:**
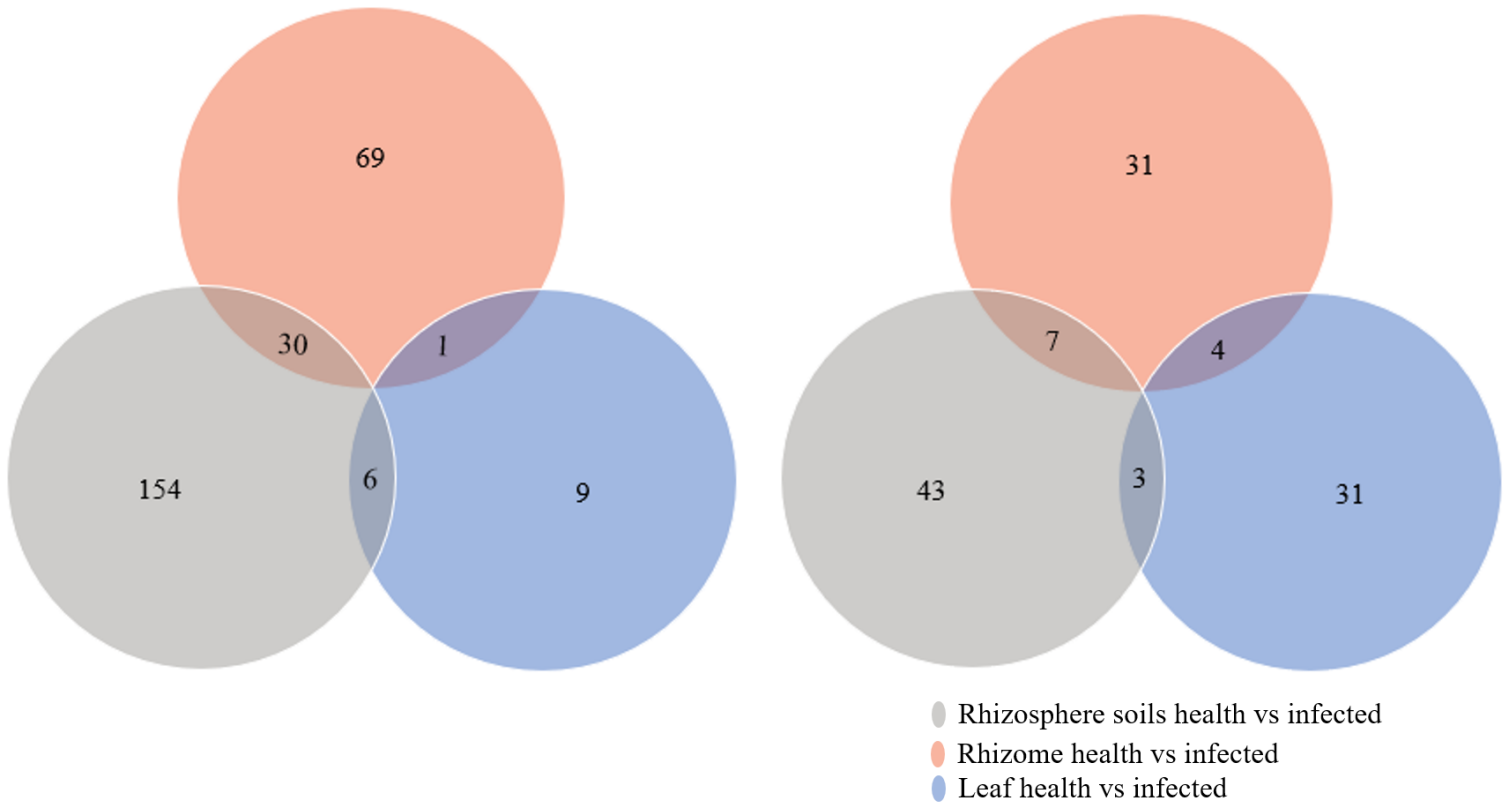
Venn diagram of the differential microbe among health and infected rhizosphere soils, rhizome, and leaf of *C. chinensis*. **(A)** Venn diagram of the differential bacteria among health and infected rhizosphere soils, rhizome, and leaf of *C. chinensis*; **(B)** Venn diagram of the differential fungi among health and infected rhizosphere soils, rhizome, and leaf of *C. chinensis*.

In order to further determine the microbes whose abundance changes lead to the disturbance of soil and plant microbial homeostasis and are related to the occurrence of *C. chinensis* root rot, the DESeq2 package in R was used to analyze the relative abundance of microbes in the rhizosphere soil, rhizomes, and leaves of healthy and diseased *C. chinensis* sample. As shown in Figure 5, the differential bacterial communities of *C. chinensis* leaf samples were relatively less, indicating that the infection had the weakest effect on the bacterial community of *C. chinensis* leaf samples. The effects of infection on the fungal community in the rhizosphere soil, rhizome, and leaf samples of *C. chinensis* were relatively comparable. However, no common intersection in differential microbial communities was found among the three types of *C. chinensis samples.* Because the rhizosphere soil and roots in space contact more closely, we speculated that the differential microbial communities shared by rhizosphere soil and rhizome samples in healthy versus infected pairwise comparison played an important role in affecting microbial homeostasis. In total, there were 30 shared genera of differential bacterial communities (Supplementary Table S3), and 7 shared genera of differential fungal communities (Supplementary Table S4). The abundance of 22 shared genera of differential bacterial communities including *Aridibacter*, *Sphingopyxis*, *Oligoflexus*, *Pseudorhodoferax*, *Polaromonas* in the infected group was higher than that in the healthy group, which may play an important role in inhibiting the fungal pathogens of *C. chinensis* (Supplementary Table S5). Subsequent screening of antagonists should focus on these genera. In terms of fungi, *Clonostachys*, *Plenodomus*, and unclassified_Ceratobasidiaceae significantly enriched in the infected group, they may promote disease occurs or were the potential pathogen of *C. chinensis* root rot. While the abundance of *Meliniomyces*, *Oidiodendron*, *Xenochalara* and unidentified_Glomeraceae in healthy groups was significantly higher than that in infected groups, and they may play a certain role in inhibiting the root rot of *C. chinensis* (Supplementary Table S6).

### 3.7. Correlation analysis between core microorganisms and soil physical and chemical factors

**Figure 6:**
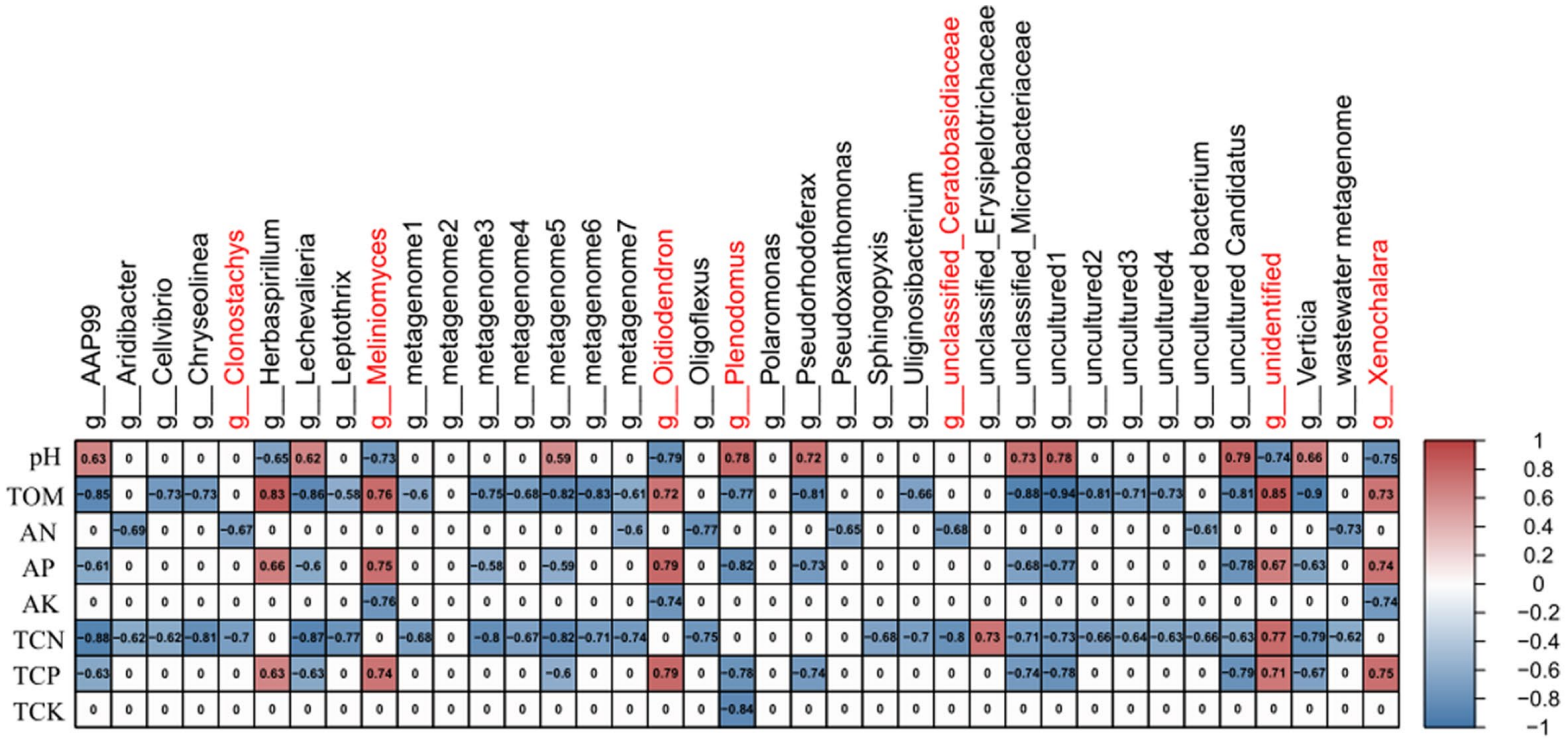
Correlation between soil physicochemical factors of rhizosphere soil and core microorganisms. Red font is marked as fungi; black font is marked as bacteria; red represents positive correlation; blue represents negative correlation in table.

Since the rhizosphere soil is in close contact with the rhizome, the root is the main site for the interaction between plants and microorganisms (Lundberg et al., 2012), we performed the correlation analyzes between soil physicochemical factors and the differential microbes (30 bacteria and 7 fungi) shared by the rhizosphere soil samples and rhizome samples (in Figures 5A,B). First, based on Bary-curtics distance, the Mantel analysis was performed using Vegan package in R. The results showed that the core differential microbial structure was significantly correlated with the structure of soil factors (Significance = 0.021; Supplementary Table S7). In order to explore the specific one-to-one correlation between the core differential microbes and the physicochemical properties of the rhizosphere soil, the Spearman correlation analysis was conducted using WGCNA package in R. As shown in Figure 6, a significant correlation was observed between the soil factors and the differential microbes (healthy vs. infected) shared by rhizosphere soil samples and rhizome samples. It could also be seen from the Figure 6 that fungi and rhizosphere soil physicochemical factors were mostly positively correlated, while bacteria and rhizosphere soil physicochemical factors were mostly negatively correlated. Physicochemical properties such as pH, organic matter, total nitrogen, total phosphorus, available nitrogen, and available phosphorus were relatively strongly correlated with microbial communities, while total potassium and available potassium were weakly correlated with microbial communities (Figure 6).

## 4. Discussion

Soil microbial communities are widely considered as an integrated component of soil quality due to their important roles in many ecological processes (Fierer et al., 2012). Soil microbial community richness and diversity play a crucial role in soil quality, function, and productivity, and soil properties may be an important factor controlling microbial community structure (Lauber et al., 2008; Song et al., 2018). Spearman correlation analysis (based on the WGCNA package) showed that pH, organic matter, total nitrogen, total phosphorus, available nitrogen, and available phosphorus were significantly correlated with microbial communities. This result is consistent with previous study report that physicochemical properties such as soil fertility can significantly affect soil microbial community structure (Bai et al., 2018). The differential microbes in diseased versus healthy *C. chinensis* comparison were strongly correlated with total nitrogen, total phosphorus, available nitrogen, and available phosphorus, but weakly correlated with total potassium and available potassium, suggesting that *excessive* nitrogen and phosphate were applied in *Coptis chinensis* planting area, but relatively less potassium was applied. Fungi and rhizosphere soil physicochemical factors were mostly positively correlated, while bacteria were mostly negatively correlated with rhizosphere soil physicochemical factors, indicating that excessive fertilization might also be one of the important reasons for the serious fungal disease of *C. chinensis*.

In this study, we found that infection with root rot disease had a great impact on the diversity and abundance of microbial communities in the rhizosphere soil, rhizome, and leaf samples of *C. chinensis*. It is well known that many microorganisms can induce or promote plant defense against various pathogens (Maksimov et al., 2011; Pieterse et al., 2014). The reduction in microbial diversity and abundance has been recognized as a major threat to ecosystems and a major cause of soil function loss, which is also the main cause of continuous cropping obstacles (Singh et al., 2014; Maron et al., 2018). In this study, infection with root rot did not significantly reduce microbial diversity and abundance, but infection with root rot significantly altered the fungal and bacterial community structure in the rhizosphere soil, rhizome, and leaf samples of *C. chinensis*. In addition, root rot infection of *C. chinensis* severely disrupted the ecological balance of the original bacterial and fungal microbiomes of healthy *C. chinensis*, and root rot infection significantly inhibited the interaction of endophytic fungi and bacteria in *C. chinensis* Zhang et al. (2017) found that Huanglongbing infection led to a decrease in the relative abundance of the microbiomes enriched on the surface of citrus roots and the expression activity of specific functions, thus leading to impaired plant-host-microbiome interactions. The rhizosphere surface microbes and endophyte microbes in the cotton infected with Verticillium wilt were significantly altered, and the numbers of rhizosphere fungi and endophyte fungi were significantly increased in diseased plants (Zhang et al., 2022). Beneficial microbial communities are involved in plant-pathogen interactions, and they possess the energy and potential to effectively enhance plant growth and productivity. The dominant microbiotas in citrus rhizosphere microorganisms are *Burkholderia* and *Bradyrhizobium*, and previous experiments have shown that inoculation of *Burkholderia* strain isolated from the related microorganisms in healthy citrus roots can induce the expression of systemic resistance-related genes in the inoculated plants, thus functioning in resisting citrus Huanglongbing (Zhang et al., 2017). Our sequencing data also showed that *Burkholderia* and *Bradyrhizobium* were dominant in the rhizome samples of *C. chinensis*, and the relative abundance of *Burkholderia* in healthy rhizome samples was significantly higher than that in diseased rhizome samples. The pathogen of *C. chinensis* root rot disease destroyed the ecological balance of *Burkholderia* during the infection process, which indirectly indicated that *Burkholderia* might play a certain role in resisting *C. chinensis* root rot. Plant pathogens need to break through the barrier of microorganisms on the rhizosphere surface to invade plants, which provides a new perspective for plant protection. Namely, crops could be protected from diseases by building a healthy microbial system. Cucumber plants susceptible to Fusarium tend to enrich beneficial microorganisms such as *Comamonadaceae* and *Xanthomonadaceae* to control Fusarium wilt [37]. Previous studies have found that leaf fall of healthy adult cacao (*Theobroma cacao*) brings the intact microbiome to the ground, thereby protecting seedlings from disease (Christian et al., 2017). Li et al. (2021) have analyzed the characteristics of the microbial community in the rhizosphere and root of *Astragalus membranaceus* infected with root rot disease by sequencing, and have constructed an artificial microbial community based on the significantly enriched bacteria in the rhizosphere of *A. membranaceus* infected with root rot disease. This artificial microbial community can inhibit the growth of pathogens and trigger the induced systemic resistance (ISR) of the diseased *A. membranaceus*, thus reducing the incidence of *A. membranaceus* root rot disease (Li et al., 2021). In our sequencing data, *Bradyrhizoloium* sp., *Acidibacter* sp., and *Streptemyce*s sp. were significantly enriched in the diseased rhizome samples. Although these bacterial communities were not the same as those enriched in *A. membranaceus* root rot samples, these bacterial communities also will be the focus of our follow-up research. Potato common scab disease affects the rhizosphere soil microbial community structure, which in turn is significantly associated with the expression of Scab toxin gene *txtAB* (Shi et al., 2019). All these findings indicate that plant roots can selectively accumulate beneficial microorganisms and directly inhibit pathogens to improve crop yield. In addition, the healthy microbial system of plants can also be artificially constructed to improve the induced systemic resistance of plants or to directly participate in the resistance to pathogens. The destruction of the microecological balance between the rhizosphere soil microbiome and the host endogenous microbiome may also be an important reason for the aggravation of disease damage and the difficulty of prevention and control.

This study focused on the influence of root rot infection on rhizosphere microbial community structure of *C. chinensis*, but the specific process of this influence was not analyzed. Was it the pathogen that affected the root exudates of *C. chinensis*, then affected the rhizosphere soil microbial community structure, and then leads to changes in the microbial community structure of rhizome and leaf of *C. chinensis*? Or does the pathogen directly produce some compound that stresses on the structure of the microbial community? These problems were urgent for us to try to solve. In addition, *Aridibacter*, *Sphingopyxis*, *Oligoflexus*, *Pseudorhodoferax*, *Polaromonas Meliniomyces*, *Oidiodendron*, and *Xenochalara* play an important role in inhibiting the root rot of *C. chinensis*. However, these microorganisms have not been isolated, identified and functionally verified. Later work will focus on the isolation and identification of rhizosphere soil microorganisms, the verification of bacteriostatic and growth-promoting functions, and the construction of synthetic bacteria for root rot prevention and control.

## 5. Conclusion

In this study, we confirmed that infection with root rot would destroy the ecological balance of the microbiomes in the rhizosphere soil, rhizome and leaf samples, and that some microbiotas were strongly correlated with soil physicochemical factors. Unfortunately, the sequencing data failed to reveal the main pathogen for *C. chinensis* root rot disease. It is speculated that *C. chinensis* root rot may be caused by the combined infection of multiple pathogens. Root rot infection destroyed the ecological balance of the original microbiomes in the rhizosphere soil, rhizome, and leaf samples of *C. chinensis. Bradyrhizoloium* sp., *Acidibacter* sp., and *Streptemyce*s sp. which would be the focus of following microecological regulation research, were significantly enriched in the diseased rhizome samples. These results provide direction for the use of micro-ecological regulation methods to prevent and control the root rot of *C. chinensis*.

## Data availability statement

The datasets presented in this study can be found in online repositories. The names of the repository/repositories and accession number(s) can be found in the article/Supplementary material.

## Author contributions

TT, FW, TM, JG, XG, and YD performed the experiments and analyzed the data. GF, HK, GS, and JY designed the experiments, supervised the study, evaluated the data, and wrote and revised the manuscript for publication. All authors contributed to the article and approved the submitted version.

## Funding

This work was supported by the central government guides local science and technology development fund projects (2022BGE256), Hubei Technology Innovation Center for Agricultural Sciences-Key Research and Development Project of Science and Technology (2020–620–000-002-04).

## Conflict of interest

The authors declare that the research was conducted in the absence of any commercial or financial relationships that could be construed as a potential conflict of interest.

## Publisher’s note

All claims expressed in this article are solely those of the authors and do not necessarily represent those of their affiliated organizations, or those of the publisher, the editors and the reviewers. Any product that may be evaluated in this article, or claim that may be made by its manufacturer, is not guaranteed or endorsed by the publisher.

## Footnotes

^1^ https://ngdc.cncb.ac.cn/gsa/

^2^ https://docs.qiime2.org/2020.11/tutorials/

^3^ http://greengenes.secondgenome.com/

^4^ https://unite.ut.ee/
