# Supplementary figures and images for: Coptischinensis Franch root rot infection disrupts microecological balance of rhizosphere soil and endophytic microbiomes
Source: Front Microbiol. 2023 May 25;14:1180368. doi: 10.3389/fmicb.2023.1180368 (PMC10248259; doi:10.3389/fmicb.2023.1180368)

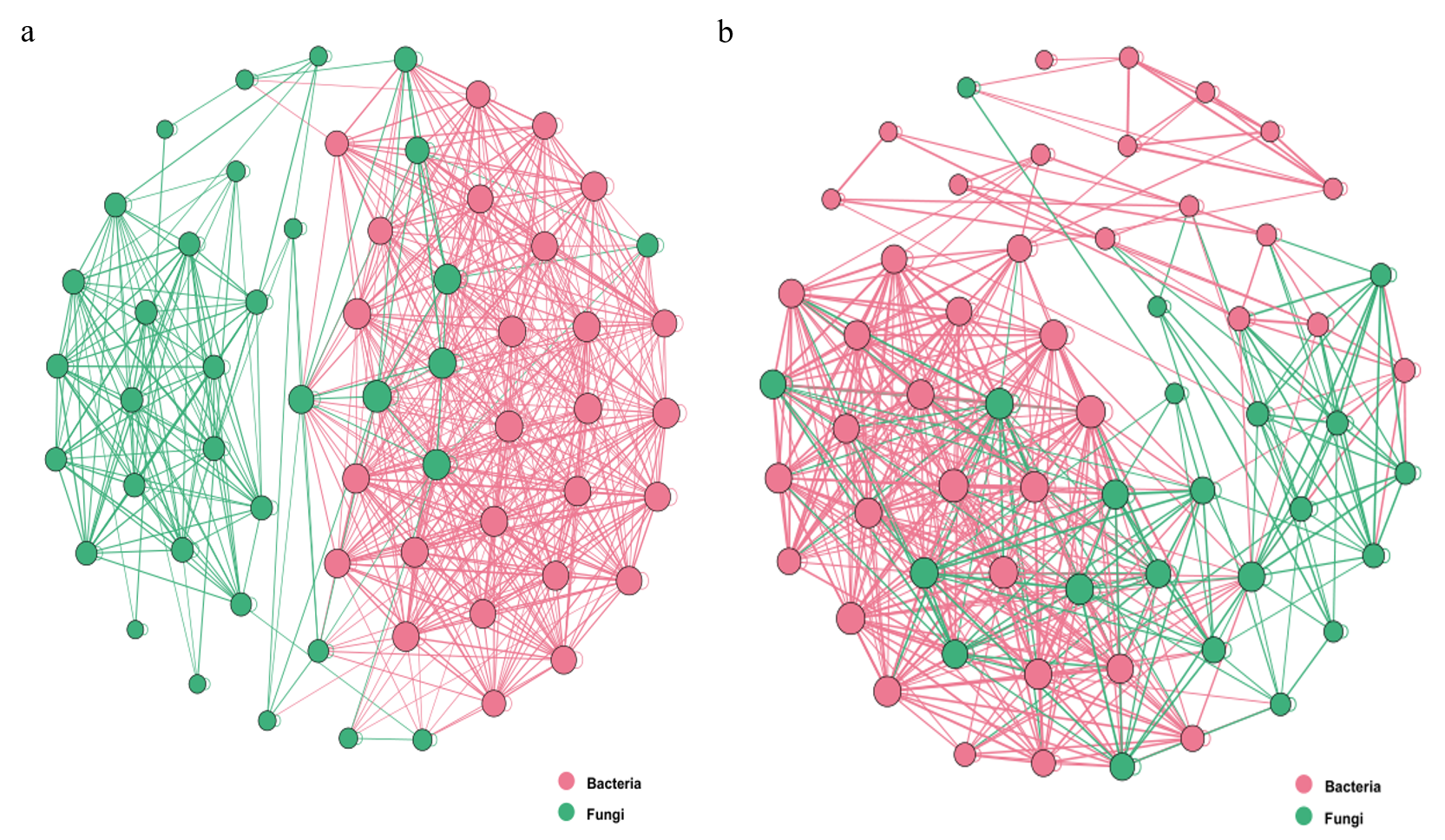

Supplement: Supplementary file 8 [file Image_1.tif]
